# Traumatic bleeding and mortality in mice are intensified by iron deficiency anemia and can be rescued with tranexamic acid

**DOI:** 10.1016/j.rpth.2024.102543

**Published:** 2024-08-08

**Authors:** Bilgimol Chumappumkal Joseph, Tro Sekayan, Nicca Falah, Richard F.W. Barnes, Veronica Flood, Juan A. De Pablo-Moreno, Annette von Drygalski

**Affiliations:** 1 Department of Medicine, Division of Hematology/Oncology, University of California San Diego, La Jolla, California, USA; 2 Versiti Blood Research Institute, Department of Pediatrics, Medical College of Wisconsin, Milwaukee, Wisconsin, USA; 3 Department of Genetic, Physiology and Microbiology, Biology School, Complutense University of Madrid, Madrid, Spain

**Keywords:** anemia, bleeding, blood loss, coagulopathy, surgery, tranexamic acid, trauma, fibrinolysis

## Abstract

**Background:**

Clinical evidence suggests that anemia exacerbates traumatic bleeding and worsens outcomes.

**Objectives:**

To study the influence of iron deficiency anemia on traumatic bleeding, coagulopathy, and mortality.

**Methods:**

C57BL/6J mice received an iron-deficient diet (8 weeks; ±1 mg intraperitoneal iron dextran 2 weeks before trauma). Control mice received a normal diet. Iron deficiency anemia was confirmed by hematocrit, red cell indices, and liver iron. Mice received saline or tranexamic acid (TXA; 10 mg/kg) just before liver laceration. Blood loss, coagulopathy (activated partial thromboplastin time, factor [F]II, FV, FVIII, FX, and fibrinogen), D-dimer, thrombin-antithrombin complexes, and plasmin-alpha-2-antiplasmin complexes were analyzed at 15 and 60 minutes, and a cytokine panel was performed at 60 minutes and 6 hours after trauma. Survival was monitored for 7 days.

**Results:**

Compared with nonanemic mice, anemic mice had lower hematocrit and hepatic iron content. Anemic mice experienced higher blood loss compared with nonanemic mice, which was reduced by TXA. Both groups developed traumatic coagulopathy characterized by activated partial thromboplastin time prolongation, thrombin-antithrombin complex formation, and depletion of FV, FVIII, and fibrinogen. TXA corrected the coagulopathy. However, plasmin-alpha-2-antiplasmin complex formation and D-dimers, markers of fibrinolysis, were higher in anemic mice and were not corrected by TXA. Seven-day survival was low in anemic mice, and rescued by TXA, but high in nonanemic mice without additional improvement by TXA. Among cytokines, only interleukin-6 increased, which was prevented by TXA most notably in anemic mice.

**Conclusion:**

These observations provide first and critical proof-of-principle evidence that anemia accelerates traumatic bleeding and increases mortality, which could be rescued by anemia correction (parenteral iron) or periprocedural TXA.

## Essentials

• Pretraumatic and presurgical anemia may be linked to increased hemorrhage and poor outcomes.
• We studied traumatic hemorrhage and effects of tranexamic acid (TXA) in anemic mice.
• TXA rescued anemia-associated bleeding and mortality but did not rescue excessive fibrinolysis.
• There is a need to study benefits of prophylactic TXA on surgical outcomes of anemic subjects.

## 1 Introduction

Preoperative anemia is a common finding in 10% to 60% of patients [1] and is an independent risk factor for postsurgical mortality [2,3]. Moreover, hematocrit and mortality risk exhibit a negative linear relationship [4]. Fewer data are available for outcomes related to preexisting anemia in the setting of trauma (“pretraumatic” anemia) because trauma is an unplanned acute event, with limited availability of pretraumatic blood values. However, the Committee on Emergency Medicine, Intensive Care, and Trauma Management of the German Trauma Society published registry data of 67,595 patients to analyze the influence of anemia at the time of admission to the Emergency Department on trauma outcomes. Preexisting moderate and severe anemia (hemoglobin, ≤8 g/dL; present in 5.1% of patients) was an independent predictor of mortality, and the anemia was considered chronic in ∼25% of cases [5]. Reasons for compromised outcomes in the setting of preexisting anemia are poorly understood, but some evidence suggests that anemia is associated with a higher risk of hemorrhage [[6], [7], [8], [9], [10], [11], [12], [13]]. In turn, intraoperative hemorrhage is associated with higher mortality [[14], [15], [16], [17]], but a direct link between anemia, the likelihood of hemorrhage, and mortality has not been established.

Anemia has also been linked to an increased bleeding risk in nonsurgical conditions, such as myocardial infarction [18,19], platelet disorders [20], and postpartum hemorrhage (PPH) [21], and in association with anticoagulation for atrial fibrillation and venous thromboembolism (VTE) [[22], [23], [24]].

*In vitro* observations indicate that low red cell numbers and hemoglobin depletion may impact blood rheology, shear stress–dependent platelet activation provision of surface lipids for thrombin generation, and fibrinolysis, as recently reviewed in depth by Lassila and Weisel [25]. However, *in vivo*, it is unknown if, and to what extent, these mechanisms increase bleeding in the presence of anemia.

The lack of *in vivo* data to improve our understanding of the effects of anemia on traumatic bleeding is a knowledge gap given that anemia (mostly caused by iron deficiency) affects ∼25% to 30% of the world population [26] and that trauma is the leading cause of death in the working population globally [27,28]. While there is clinical evidence to suggest that early administration of tranexamic acid (TXA) reduces mortality due to bleeding after acute trauma [29], the efficacy of TXA in the presence of anemia is unknown.

Therefore, to better understand the influence of anemia on traumatic hemorrhaging, coagulopathy, and mortality, we employed a model of traumatic liver injury in iron-deficient anemic mice and evaluated effects of periprocedural TXA on bleeding and mortality.

## 2 Methods

### 2.1 Mouse models

All animal protocols were approved by the Institutional Animal Care and Use Committee of the University of California San Diego. C57BL/6J mice were obtained from Jackson Laboratory and the University of California San Diego internal breeding facility. Both male and female mice aged 8 to 10 weeks were used for experiments.

### 2.2 Mouse model of iron deficiency anemia (± iron repletion)

Starting at 3 weeks of age, mice were fed an iron-deficient diet (4 ppm iron diet [TD.80396], Envigo). Control mice were fed a normal laboratory diet or received intraperitoneal iron (1 mg; MWI) 6 weeks after the start of the iron-deficient diet. At 8 to 10 weeks, blood was collected via submandibular venipuncture into EDTA-containing polypropylene microtubes (Becton Dickinson). A complete blood count was carried out using a Hemavet 950FS Multi-Species Hematology System (Drew Scientific) programmed for mouse blood parameters. Hematocrits were spun directly. Whole blood smears were prepared using 5 μL of blood and stained with Wright-Giemsa (Sigma-Aldrich) using standard protocols. Slides were mounted using Refrax mounting medium (Anatech Ltd) and cover-slipped. Digital photomicrographs were taken using an Olympus BH2 microscope and an oil immersion lens at 100×, equipped with an Olympus MicroFire digital camera (Olympus Corporation).

### 2.3 Mouse model to induce traumatic bleeding by liver laceration

A liver laceration model was used to cause internal hemorrhage as described previously [30]. Mice (∼10 weeks old) were anesthetized with 1.5% isoflurane and 2 L/min of O_2_ and secured in the supine position on a metal board. Mice were given 0.5 mg/kg of buprenorphine-SR (Zoopharm) or 3.25 mg/kg of Ethiqa (Fidelis Animal Health) subcutaneously before surgery. The abdominal cavity was exposed by midline laparotomy. Three triangle-cut preweighed pieces of filter paper (∼0.035 g each) were inserted into the abdominal cavity, and ∼75% of the left lobe of the liver was removed with sharp scissors. The abdominal skin was securely closed using wound clips (AutoClip kit, Fine Science Tools) to avoid leakage of blood. Mice were monitored for the duration of the experiment (15- and 60-minute time points). At the end of the experiment, wound clips were removed and filter papers were collected. A fourth piece of preweighed filter paper was used to absorb any remaining blood in the abdominal cavity. Blood loss was determined by weighing the blood-soaked filter papers and was expressed in microliters per gram of the mouse body weight. Blood loss in anemic mice was compared with blood loss in nonanemic mice [31]. A scheme is provided in Supplementary Figure S1.

### 2.4 Survival study

For the survival model, blood loss was determined 60 minutes after liver laceration. At the end of the 60-minute period, the abdominal skin was sutured using sterile conditions. Mice received 400 μL of saline (Hesperia) subcutaneously prior to being returned to their cages, followed by additional daily injections of 400 μL of saline for the first 3 days. A second dose of 0.5 mg/kg of buprenorphine-SR (sustained-release) or 3.25 mg/kg of Ethiqa was injected subcutaneously after 72 hours to alleviate pain. Mice were observed continuously for the first 24 hours, followed by cage checks every 6 hours on day 2, and then daily thereafter.

### 2.5 TXA administration

TXA (AuroMedics) at a dose of 10 mg/kg was diluted in 0.9% sterile saline for injection and administered as a single bolus by retro-orbital injection 5 minutes before liver laceration. Control mice for both nonanemic and anemic mice received an equal volume of 0.9% sterile saline (100 μL).

### 2.6 Blood collection and processing

At the end of the 15-minute (anemic and nonanemic) or 60-minute (anemic, nonanemic, and iron-replete) experimental period, blood was collected by cardiac puncture or through retro-orbital access into 3.8% sodium citrate. A 9:1 blood-to-anticoagulant ratio was used for measurements of clotting factor activity in plasma. In samples with a hematocrit of <35, the anticoagulant volume was adjusted by using the formula *C* = (1.85 × 10^−3^) (100 − Hematocrit) (*V*_Blood_), where *C* is the volume of sodium citrate in milliliters, *V* is the volume of whole blood, and hematocrit in percentage, 1.85 × 10^−3^ is a constant (taking into account the citrate volume, blood volume, and citrate concentration) [32]. Samples were centrifuged at 2000*g* for 10 minutes and 13,500*g* for 5 minutes to obtain the platelet-poor plasma and used for blood coagulation tests and other analyses. All plasma samples were stored at −80 °C until analysis.

### 2.7 Determination of coagulation factor activity levels

All clotting times were recorded using an ST4 coagulometer (Diagnostica Stago). Activated partial thromboplastin time (aPTT) was determined by mixing 25 μL mouse plasma with 25 μL aPTT reagent (Diagnostica Stago), followed by the addition of 25 μL CaCl_2_ (25 mM) in 2-(4-(2-hydroxyethyl)-1-piperazinyl)-ethanesulfonic acid-buffered saline (HBS; 20 mM 2-(4-(2-hydroxyethyl)-1-piperazinyl)-ethanesulfonic acid, 147 mM NaCl, 3 mM KCl, pH 7.4) after 3 minutes at 37 °C [33]. To determine the activity levels of various coagulation factors, murine plasma (5 μL) was mixed with factor (F)II-, FV-, or FX-deficient plasma (Enzyme Research Laboratories; 20 μL) and HBS with 0.5% bovine serum albumin (25 μL; Sigma-Aldrich) and incubated at 37 °C for 1 minute. The clotting time was recorded following the addition of Innovin (DADE Behring). To determine FVIII activity levels, murine plasma (5 μL) was mixed with FVIII deficient plasma (20 μL; Enzyme Research Laboratories) and aPTT reagent and incubated at 37 °C for 3 minutes following the addition of 25 μL CaCl_2_ (25 mM) in HBS 0.5% bovine serum albumin. FVIII chromogenic assays were performed using the DiaPharma kit (DiaPharma) according to the manufacturer’s protocol. Fibrinogen concentration was measured using the Clauss method [34]. von Willebrand factor (VWF) levels were measured by enzyme-linked immunosorbent assay (ELISA) using anti-VWF antibody (rabbit anti-human VWF polyclonal antibody, Dako) as previously described [35] and quantitated at 405 nm using the Versa Max microtiter plate reader (Molecular Devices). VWF levels for iron-replete mice were measured at Versiti Blood Research Institute, Milwaukee, Wisconsin. von Willebrand factor antigen (VWF:Ag) was measured in the clinical laboratory at Blood Center of Wisconsin by an ELISA using 2 monoclonal antibodies (AVW-1 and AVW-5, Versiti Blood Research Institute) for capture and an horseradish peroxidase–conjugated rabbit polyclonal anti-VWF antibody for detection (Dako North America) [36].

### 2.8 Determination of plasma biomarkers

Thrombin-antithrombin complex (TAT) and plasmin-alpha-2-antiplasmin (PAP) complex levels were determined using the Enzygnost (Siemens Healthcare) and Novus Biological (Novus Biologicals) kits as per the manufacturer’s instructions. Total plasma D-dimer was measured using a mouse D-Dimer ELISA kit (Thermo Fisher Scientific). To evaluate the interference of D-dimer on aPTT analysis, plasma samples collected at 60 minutes were serially diluted with baseline plasma and aPTT was performed.

### 2.9 Nonheme liver iron concentration

Iron concentration was measured with a colorimetric iron SL assay kit (Sekisui Diagnostics) as previously described [37]. In brief, ∼75% of the liver was homogenized, and 75 μL from the homogenate was weighed and incubated in 1125 μL protein precipitation solution (0.53 N HCl and 5.3% trichloroacetic acid) at 100 °C for 60 minutes. After centrifugation to remove tissue debris, the supernatant was analyzed for iron concentration using Iron SL assay (Sekisui Diagnostics). Serial dilutions of Iron AA standard (RICCA chemical companyIron AA - iron atomic absorption standard) (RICCA Chemical Company) were used to generate the standard curve normalized to tissue weight.

### 2.10 Plasma cytokine analysis

Cytokine content of plasma was determined by a commercially available ELISA kit (14-Plex Q-Plex mouse cytokine inflammation high sensitivity kit, Quansys Biosciences). Interleukin (IL)-1 alpha, IL-1 beta, IL-2, IL-3, IL-4, IL-6, IL-10, IL-12p70, IL-17, monocyte chemoattractant protein 1, macrophage inflammatory protein 1 alpha, granulocyte-macrophage colony–stimulating factor, tumor necrosis factor alpha, and regulated on activation normal T cell expressed and secreted were measured at 60 minutes and 6 hours after liver laceration. The assay was performed according to the manufacturer’s instructions.

### 2.11 Statistical analysis

The sample sizes were not normally distributed. Therefore, data were expressed as medians, and groups were compared using the nonparametric Mann–Whitney U-test. The Bonferroni adjustment for multiple comparisons was applied when necessary. Statistical analyses were performed using GraphPad Prism Software (GraphPad Software). Survival was analyzed using Kaplan–Meier survival curves and was compared using the Mantel–Cox log-rank test.

## 3 Results

### 3.1 Effects of the iron-deficient diet on complete blood counts, hepatic iron content, and body weight

**Figure 1:**
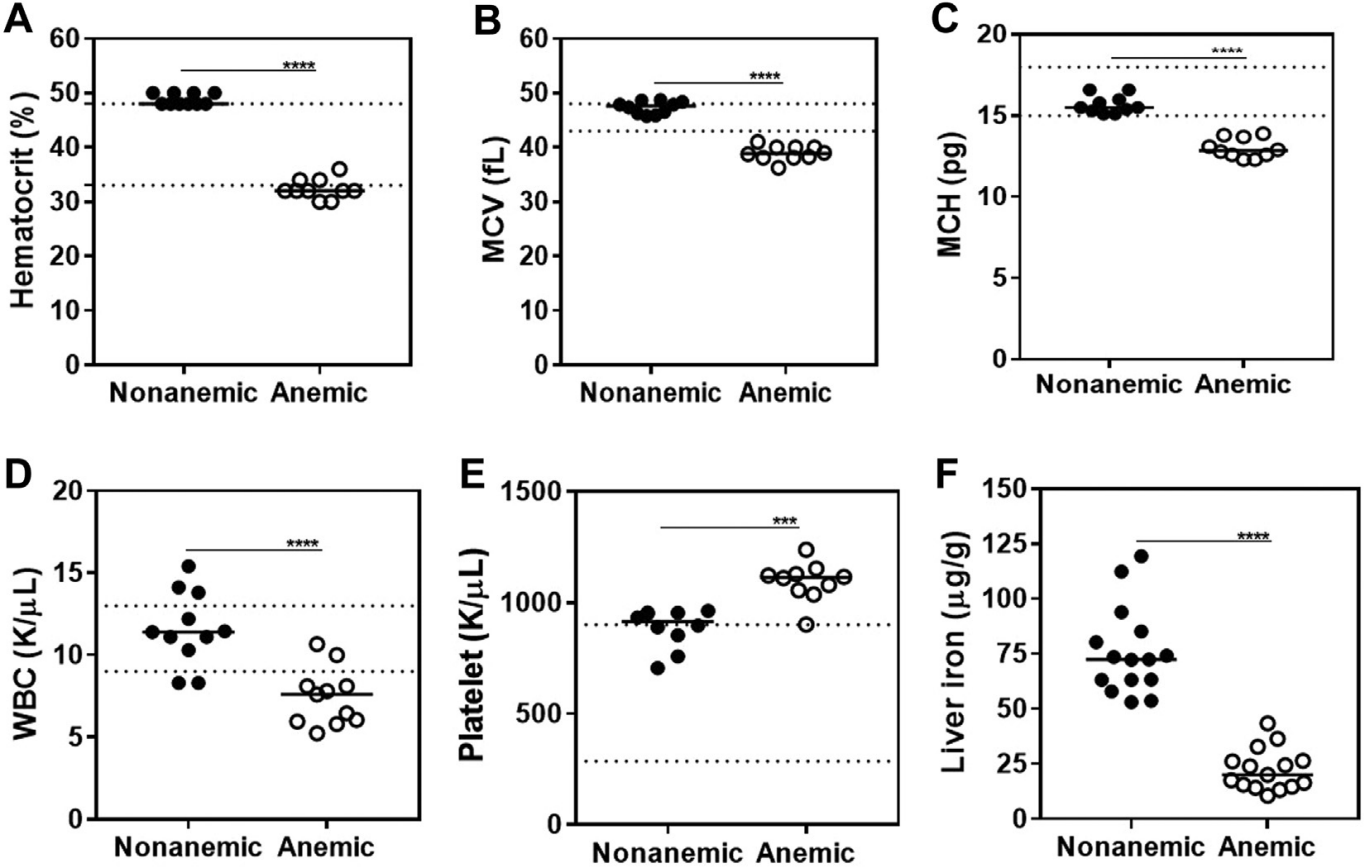
Complete blood count and liver iron content in iron-deficient mice. Prior to liver laceration, complete blood count and liver iron content were analyzed in mice fed with an iron-deficient diet. Results were compared with those of control mice fed with a normal diet. (A) Hematocrit, (B) mean corpuscular volume (MCV), (C) mean corpuscular hemoglobin (MCH), (D) white blood cell (WBC), (E) platelets, and (F) liver iron (*n* = 10-15). Samples were compared with nonparametric Mann–Whitney U-test. The horizontal bars represent the median. The area between the broken lines marks the normal range for C57Bl/6 mice [38]. ∗∗∗*P* ≤ .001; ∗∗∗∗*P* ≤ .0001.

Development of iron deficiency anemia (IDA) was assessed by hematocrit and red cell indices as well as liver iron content. Mice fed with the iron-deficient diet had significantly lower hematocrit values compared with nonanemic control mice at 6 weeks of age (32% vs 48%;*P* ≤ .0001; *n* = 10-15 per group; Figure 1A) [38]. In addition, red cells of anemic mice had reduced mean corpuscular volume (38.90 fL vs 47.65 fL; *P* ≤ .0001; *n* = 10) and mean corpuscular hemoglobin concentration (12.85 pg vs 15.50 pg; *P* ≤ .0001; *n* = 10), respectively (Figure 1B, C). Representative blood smears depicting microcytic, hypochromic, and irregularly shaped red cells are shown in Supplementary Figure S2A, B. Mean white blood cell count was lower (7.6 K/μL vs 11.4 K/μL; *P* ≤ .0001; *n* = 10) and mean platelet count was higher (1113.0 K/μL vs 914.8 K/μL; *P* ≤ .001; *n* = 10) in anemic mice than in nonanemic mice, respectively (Figure 1D, E). Hepatic iron content (Figure 1F) was significantly lower in anemic than in nonanemic mice (20.04 μg/g vs 72.41 μg/g; *P* ≤ .0001; *n* = 15). Altogether, these findings demonstrated the presence of IDA in mice fed with the iron-deficient diet. Body weight determination (determined prior to liver laceration) revealed that males were on average ∼6 g heavier than females and that median weight was ∼1 g lower in anemic than in nonanemic mice (all *P* ≤ .01; *n* = 55 per group), respectively (Supplementary Figure S3). Since blood volume is proportional to body weight, blood loss was adjusted for body weight.

### 3.2 Blood loss and prevention of blood loss by TXA after liver laceration in anemic and nonanemic mice

**Figure 2:**
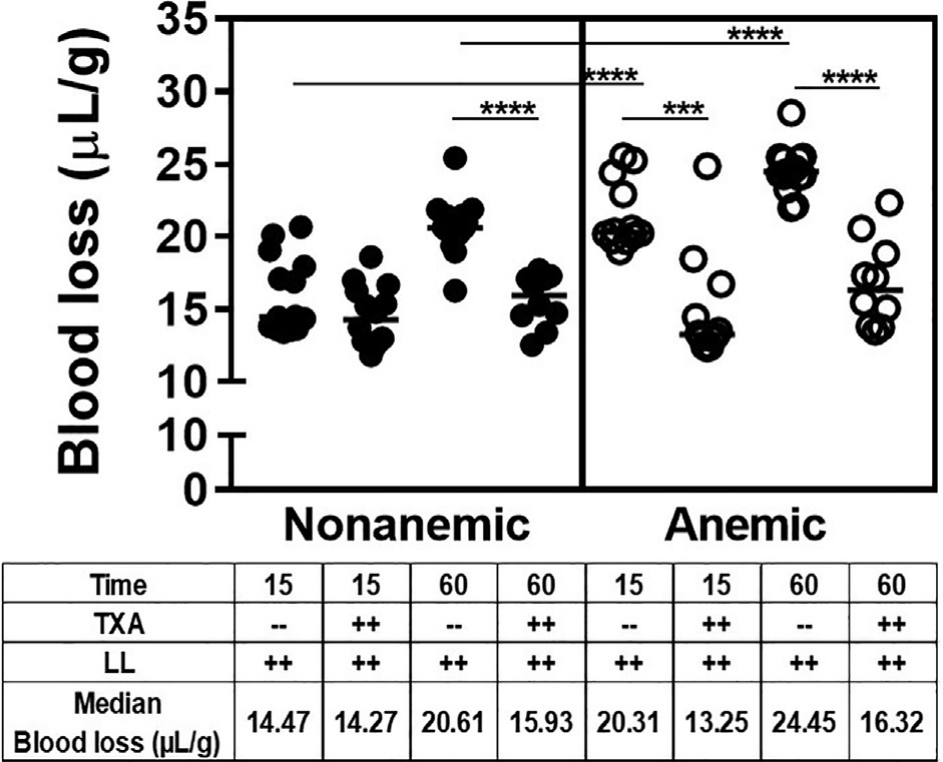
Blood loss after liver laceration (LL). Severe bleeding was induced by midline laparotomy followed by LL. Both nonanemic and anemic mice were treated prophylactically with 100 μL of saline or tranexamic acid (TXA; 10 mg/kg). Blood loss was determined in each group at the 15- and 60-minute time points (*n* = 7-21). Samples were compared with nonparametric Mann–Whitney U-test. Results were compared with baseline with horizontal bars showing the median. ∗∗∗*P* ≤ .001; ∗∗∗∗*P* ≤ .0001.

Severe bleeding, induced by laparotomy and liver laceration, resulted in significantly more blood loss in anemic mice than in nonanemic mice at both 15 and 60 minutes. At 15 minutes, median blood loss was 14.5 μL/g in nonanemic and 20.3 μL/g in anemic mice (*P* ≤ .0001; *n* = 12-14). At 60 minutes, median blood loss was 20.6 μL/g and 24.5 μL/g, respectively (*P* ≤ .0001; *n* = 15; Figure 2). Hence, the median amount of blood loss at 60 minutes in nonanemic mice (20.6 μL/g) was comparable with the median amount of blood loss at 15 minutes in anemic mice (20.3 μL/g), with continued bleeding in the anemic mice to a median of 24 μL/g at 60 minutes. Together, these findings indicate more rapid and pronounced bleeding following surgical trauma in the presence of anemia.

TXA reduced blood loss significantly in all groups of mice (with the exception of nonanemic mice at 15 minutes) to a median of ∼15 μL/g (all *P* ≤ .001; *n* = 10-14 per group). In fact, the median blood loss in nonanemic mice at 15 minutes was only 14.5 μL/g, and TXA prophylaxis did not improve bleeding below this threshold of ∼15 μL/g. (Figure 2). This suggests that prophylactic TXA is effective in uniformly reducing early blood loss in the presence or absence of anemia, but not below a distinct bleed threshold.

### 3.3 Association between iron deficiency and plasma FVIII levels

**Figure 3:**
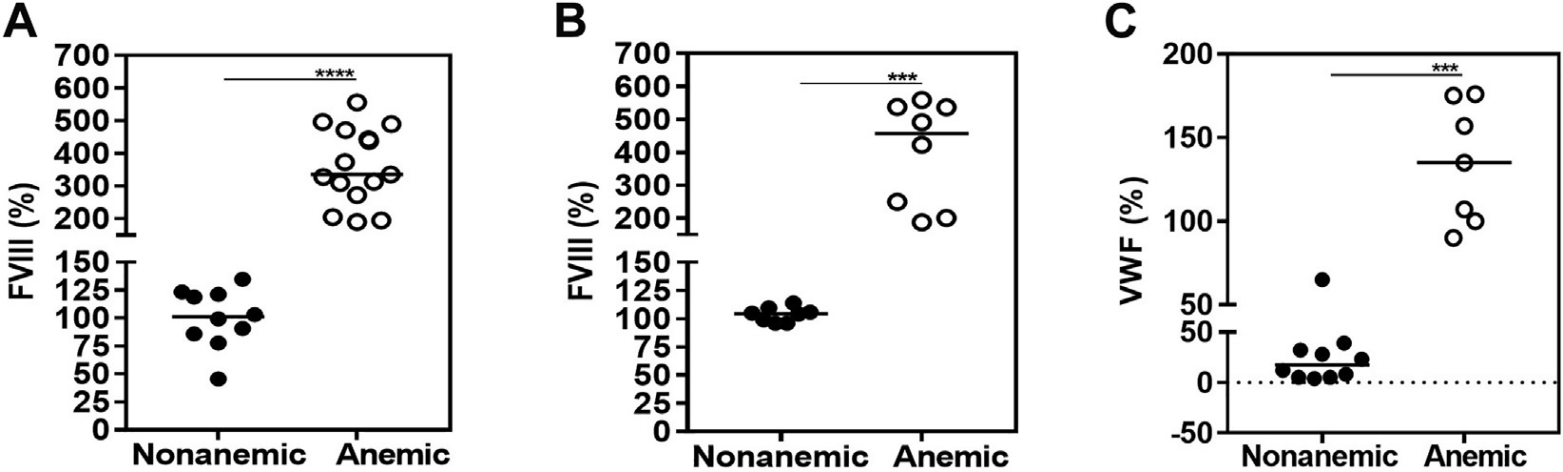
Factor (F)VIII chromogenic assay and von Willebrand factor (VWF) antigenic levels in nonanemic and anemic mice. Baseline plasma samples of nonanemic and anemic mice (6 weeks of age) were analyzed for FVIII levels by (A) 1-stage clotting assay, (B) chromogenic assay, and (C) VWF determined by enzyme-linked immunosorbent assay (*n* = 7-15 per group). ∗∗∗*P* ≤ .001; ∗∗∗∗*P* ≤ .0001.

When determining plasma clotting factor activity levels to study coagulopathy, it became evident that FVIII levels were significantly increased at baseline in anemic mice compared with nonanemic mice, using the 1-stage clotting assay (aPTT-based; 335.6% vs 101.2%; *P* ≤ .0001; *n* = 10-15) as well as the chromogenic assay (457.4% vs 104.6%; *P* ≤ .001; *n* = 8; Figure 3A, B). This finding was unexpected, has not been reported previously in mouse models of IDA, and was accompanied by elevated VWF levels (135% vs 17%; *P* ≤ .001; *n* = 10; Figure 3C).

### 3.4 Clotting parameters after liver laceration in anemic and nonanemic mice and the effects of TXA

General coagulopathy was determined by several parameters: aPTT, thrombin generation (TAT complexes), acute traumatic coagulopathy [39] (FV, FVIII, and fibrinogen) and/or disseminated intravascular coagulation (DIC; FII, FX, and fibrinogen), and fibrinolysis (fibrinogen, PAP complexes, and D-dimer). Trauma-induced coagulopathy (TIC) also known as acute traumatic coagulopathy is associated with a selective depletion of FV and FVIII mediated by activated protein C [31,39,40], whereas DIC indicates a more general nonselective clotting factor consumption state [41,42]. Additionally, we determined to what extent these parameters were influenced by TXA given its profound effect on bleed reduction.

**Figure 4:**
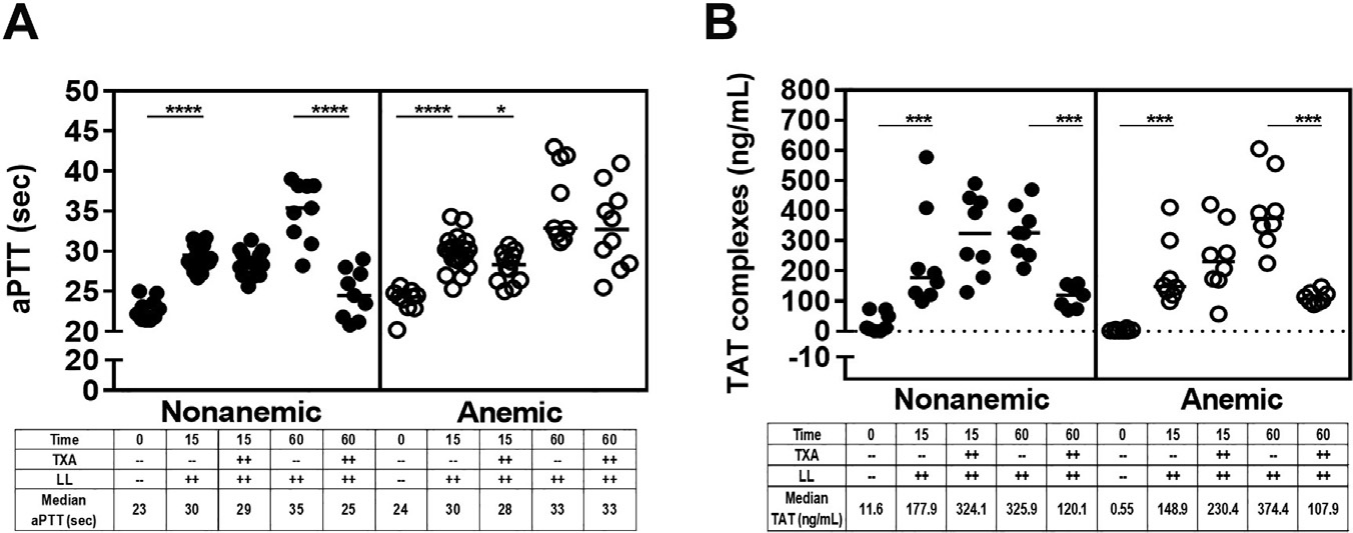
General coagulopathy after liver laceration (LL). Severe bleeding was induced by midline laparotomy followed by LL. Both nonanemic and anemic mice were treated prophylactically with 100 μL of saline or tranexamic acid (TXA; 10 mg/kg). Coagulation parameters were determined in each group at the 15- and 60-minute time points. (A) Activated partial thromboplastin time (aPTT) and (B) quantitative determination of thrombin-antithrombin (TAT) complex (*n* = 7-21). Samples were compared with nonparametric Mann–Whitney U-test. Results were compared with baseline with horizontal bars showing the median. ∗*P* ≤ .05; ∗∗∗*P* ≤ .001; ∗∗∗∗*P* ≤ .0001.

Bleeding induced by liver laceration was associated with general coagulopathy in all groups of mice. The aPTT was comparable between nonanemic and anemic mice at baseline (∼23 seconds), at 15 minutes (∼30 seconds), and at 60 minutes (∼33-35 seconds), and the prolongations from baseline were significant (all *P* ≤ .001; *n* = 9-21). TXA corrected the aPTT at 60 minutes in nonanemic mice only (Figure 4A). Also, TAT complexes increased significantly in nonanemic and anemic mice compared with baseline, with doubling noted at 60 minutes compared with the 15-minute time point (all *P* ≤ .001; *n* = 8; Figure 4B). TXA decreased TAT complex formation significantly at 60 minutes in both groups (nonanemic mice: median, 326-120 ng/mL; anemic mice: 374-108 ng/mL; all *P* ≤ .001; *n* = 8). These findings suggest that trauma elicits a similar degree of coagulopathy and thrombin generation in nonanemic and anemic mice. However, while TXA resulted in near normalization of thrombin generation at 60 minutes in both groups, it shortened the aPTT only in nonanemic mice.

**Figure 5:**
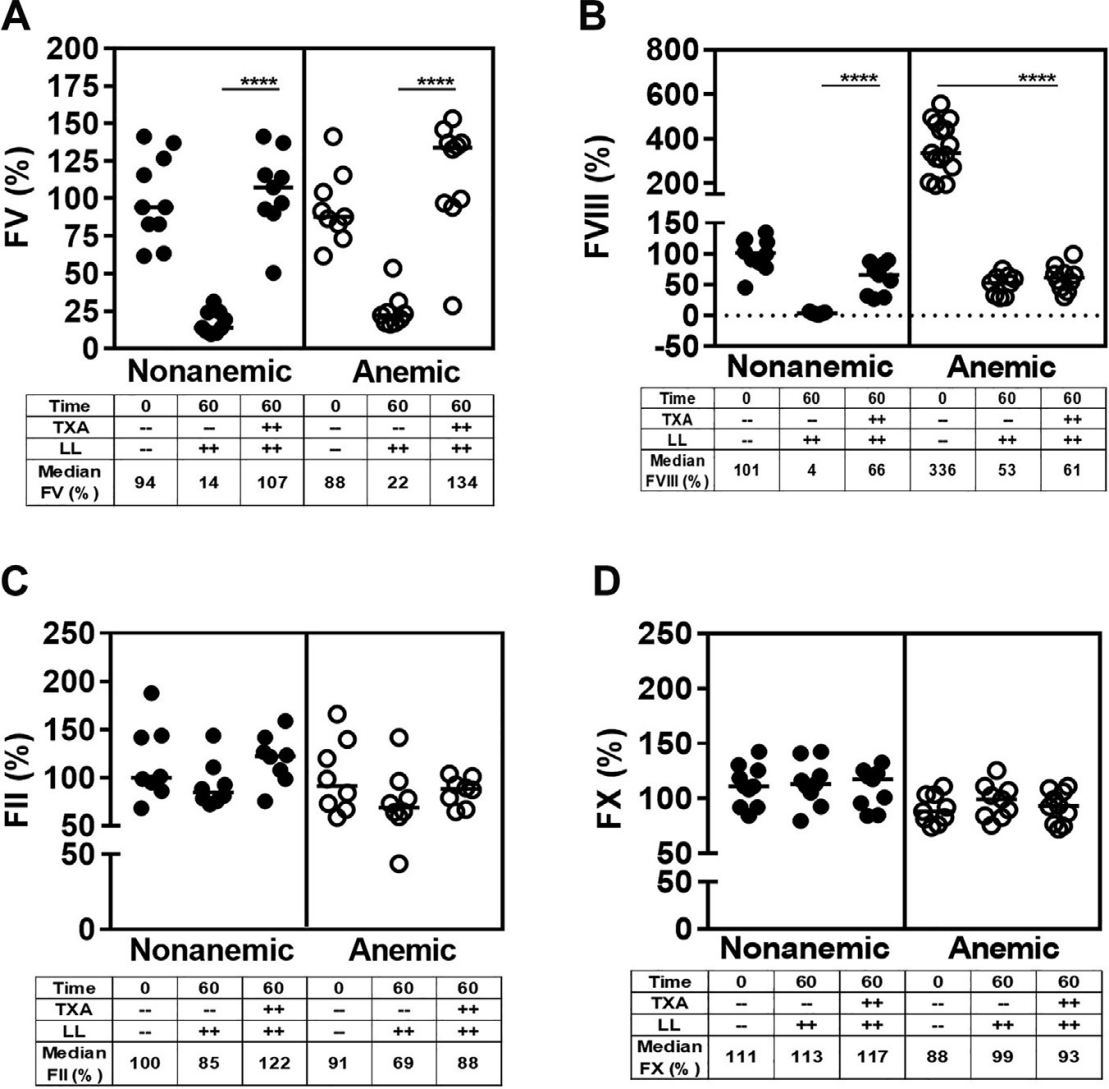
Development of trauma-induced coagulopathy or disseminated intravascular coagulation after liver laceration (LL). Development of trauma-induced coagulopathy and/or disseminated intravascular coagulation was determined after severe bleeding was induced by a midline laparotomy followed by LL in nonanemic and anemic mice. Mice were prophylactically treated with 100 μL of saline or tranexamic acid (TXA; 10 mg/kg). Factor (F)V, FVIII, FII, and FX activity levels were determined 60 minutes later. (A) FV, (B) FVIII, (C) FII, and (D) FX (*n* = 8-15). Samples were compared with nonparametric Mann–Whitney U-test. Results were compared with baseline with horizontal bars showing the median. ∗∗∗∗*P* ≤ .0001.

**Figure 6:**
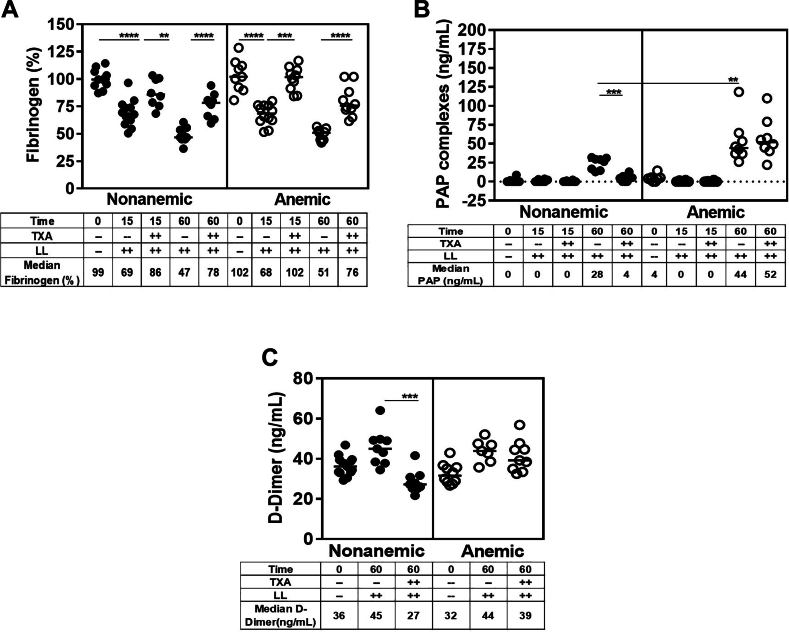
Fibrinolysis after liver laceration (LL). Plasma fibrinogen levels and fibrinolysis were determined 15 and 60 minutes after induction of severe bleeding by a midline laparotomy followed by LL in nonanemic and anemic mice. Mice were prophylactically treated with 100 μL of saline or tranexamic acid (TXA; 10 mg/kg). (A) Fibrinogen, (B) plasmin-alpha-2-antiplasmin (PAP) complex, (C) D-dimer (*n* = 8-12). Samples were compared with nonparametric Mann–Whitney U-test. Results were compared with baseline with horizontal bars showing the median. D-dimer, D fragments of the fibrin. ∗∗*P* ≤ .01; ∗∗∗*P* ≤ .001; ∗∗∗∗*P* ≤ .0001.

To elucidate if differences in clotting factor activity levels or interference with fibrin split products could explain the discrepancy of aPTT correction by TXA in anemic and nonanemic mice and to delineate the development of TIC and/or DIC, we determined FV, FVIII, FII, and FX levels and fibrinogen 60 minutes after trauma. In both nonanemic and anemic mice, traumatic bleeding was associated with a profound decrease of FV and FVIII activity (Figure 5A, B), whereas FII and FX activities were unaffected (Figure 5C, D). TXA prevented the depletion of FV activity (Figure 5A) and partially corrected FVIII levels in nonanemic mice (increase from median, 4% to 65%). In anemic mice, TXA stabilized severe FVIII depletion (median ∼61% vs baseline median 335%; *P* ≤ .0001; *n* = 9-15; Figure 5B). There was a ∼25% depletion of fibrinogen at 15 minutes in anemic and nonanemic mice, which increased to ∼50% at 60 minutes. TXA improved fibrinogen levels significantly at both time points (Figure 6A). These observations suggest that TIC, rather than DIC, dominated the coagulopathy and that the coagulopathy was similar in the presence of anemia, and could be mitigated by TXA.

There was no PAP complex formation at 15 minutes, indicating absence of measurable systemic fibrinolysis during the early phase of coagulopathy. However, at 60 minutes, PAP complex formation was present, with significantly higher levels in anemic than in nonanemic mice (median, 44 ng/mL vs 28 ng/mL; *P* ≤ .001; *n* = 8). TXA corrected PAP complex formation only in nonanemic mice (Figure 6B). These findings suggest excessive fibrinolysis in the presence of anemia that could not be improved by TXA. These observations suggested that the lack of aPTT correction with TXA at the 60-minute time point may be due to interference of fibrin split products in the aPTT assay (Figure 4A). Therefore, we studied the presence of fibrin split products (D-dimers) at 60 minutes and found that D-dimers were elevated significantly in both, nonanemic and anemic mice, compared with those in baseline. While TXA treatment suppressed D-dimer formation in nonanemic mice, it had no effects on D-dimer formation in the anemic mice (Figure 6C). These findings further strengthen the notion of increased fibrinolysis in anemic mice that could not be mitigated by TXA, as well as interference of fibrin split products with aPTT correction. To determine the latter, we performed serial plasma dilution studies (1:1 mix), which demonstrated gradual correction of the aPTT, with complete correction at a dilution titer of 1:16 in nonanemic and anemic plasma alike (± TXA). It is therefore highly plausible that the aforementioned absent correction of the aPTT was due to fibrin split product interference (Supplementary Figure S4).

### 3.5 Mortality after liver laceration in anemic and nonanemic mice and the effects of TXA

**Figure 7:**
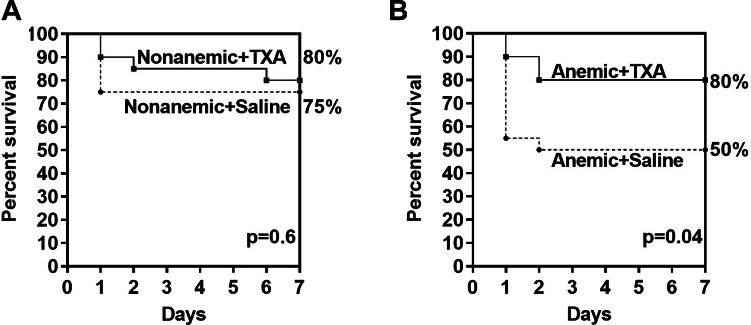
Survival after liver laceration. Seven-day survival of nonanemic and anemic mice after trauma was determined by Kaplan–Meyer analysis. Mice underwent midline laparotomy with subsequent liver laceration and were treated prophylactically with 100 μL of saline or tranexamic acid (TXA; 10 mg/kg) 5 minutes prior. After blood loss, determination was completed (60 minutes after trauma); wounds were closed; and the mice were returned to the cages, given 400 μL of saline for the first 3 days, and monitored for 7 days for survival (*n* = 20 mice per group). (A) Nonanemic mice and (B) anemic mice.

The 7-day survival rate for nonanemic mice was 75% and 80% without and with TXA, respectively. However, the 7-day survival rate for anemic mice was only 50% and improved significantly to 80% with TXA (*P* = .04; *n* = 20 per group; Figure 7A, B).

### 3.6 Anti-inflammatory effects of TXA

**Figure 8:**
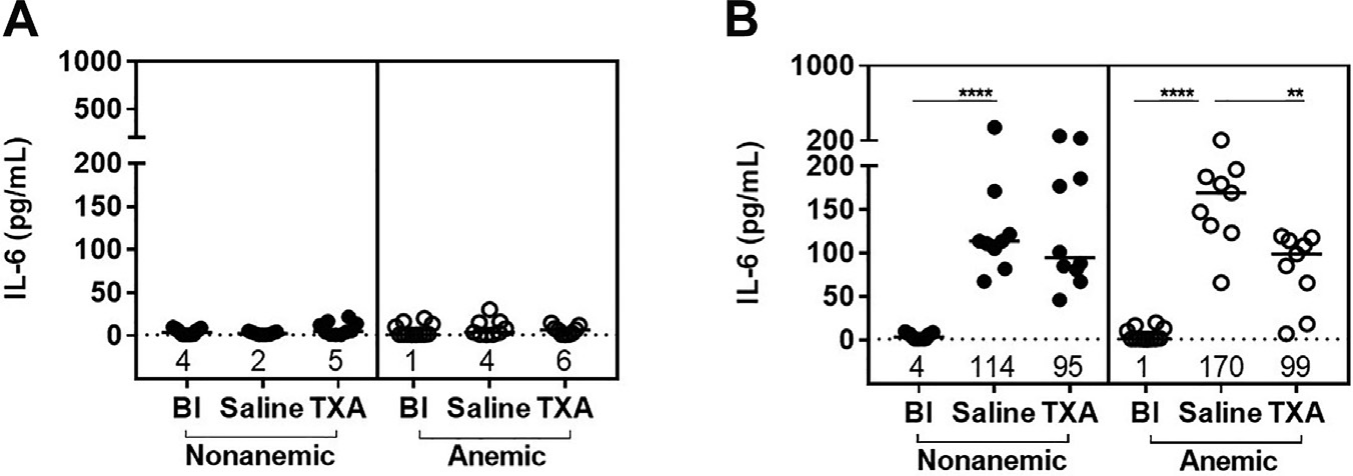
Inflammatory effects after liver laceration (LL). Plasma interleukin (IL)-6 level was determined at baseline (Bl), 60 minutes, and 6 hours after induction of severe bleeding by a midline laparotomy followed by LL in nonanemic and anemic mice, (A) 60 minutes after LL, and (B) 6 hours after LL (*n* = 9-12). Mice were prophylactically treated with 100 μL of saline or TXA (10 mg/kg). Samples were compared with nonparametric Mann–Whitney U-test. Results were compared with Bl with horizontal bars showing the median. ∗∗*P* ≤ .01; ∗∗∗∗*P* ≤ .0001.

To investigate if TXA exerts anti-inflammatory effects, we quantified 14 cytokines (IL-1 alpha, IL-1 beta, IL-2, IL-3, IL-4, IL-6, IL-10, IL-12p70, IL-17, monocyte chemoattractant protein 1, macrophage inflammatory protein 1 alpha, granulocyte-macrophage colony–stimulating factor, tumor necrosis factor alpha, and regulated on activation normal T cell expressed and secreted) in nonanemic and anemic plasma at baseline, 60 minutes, and 6 hours after liver laceration, which is the time point farthest from surgery prior to the start of mortality. Only 1 of the 14 cytokines (IL-6) was modulated by TXA. The extent of the differences between anemic and nonanemic mice was confined to changes in IL-6. IL-6 levels at 60 minutes were similar to baseline in both groups (Figure 8A). However, at 6 hours, IL-6 was significantly increased compared with baseline in anemic and nonanemic mice. This increase appeared more pronounced in anemic mice (median: anemic, 1 pg/mL vs 170 pg/mL; *P* ≤ .0001; nonanemic, 3.7 pg/mL vs 114 pg/mL; *P* ≤ .0001; *n* = 9-13). TXA reduced IL-6 in both groups, anemic and nonanemic, to a median ∼95 pg/mL, which was significant in the anemic group (median, 170-99 pg/mL; *P* ≤ .01; *n* = 9; Figure 8B).

### 3.7 Effects of iron repletion on bleeding, coagulopathy, and survival

Parenteral iron repletion of anemic mice 2 weeks prior to liver laceration corrected the IDA and normalized hematocrit (with improved mean corpuscular volume) and platelet and white blood cell counts (*n* = 14-25; Supplementary Figure S5A–E). Liver iron content exceeded baseline measurements of control (nonanemic) mice (median, 20 μg/g vs 155 μg/g; *P* ≤ .0001; *n* = 20-22; Supplementary Figure S5F). Blood loss at 60 minutes after liver laceration was significantly lower than that in nonanemic mice (15.8 μL/g vs 20.6 μL/g; *P* ≤ .0001; *n* = 15-28) without further bleed improvement by TXA below the threshold of ∼15 μL/g (Supplementary Figure S6A).

Posttraumatic prolongation of the aPTT as well as reductions in FV, FVIII, and fibrinogen at 60 minutes were comparable in iron-replete and control mice, and were all corrected by TXA (Supplementary Figure S6B–E). Iron repletion decreased FVIII levels observed in anemic mice (median, 457.4%-124.5%; *P* ≤ .001; *n* = 8-9), which approximated FVIII levels in control mice. Similarly, iron repletion decreased VWF levels (median, 140%-92%; *P* ≤ .01; *n* = 9-10), although levels were still higher than in control mice (median, 92%-20%; *P* ≤ .001; *n* = 10; Supplementary Figure S7A, B). Similar to control mice, FII and FX levels were not affected (Supplementary Figure S8A, B).

The seven-day survival rate was also comparable between iron-replete and control mice (∼75%), and the administration of TXA did not result in further improvement (Supplementary Figure S9A, B). These findings suggest that correction of IDA can reduce traumatic bleeding and mortality.

Similar to control mice, IL-6 was significantly increased at 6 hours compared with that in baseline in iron-replete mice (median control, 3.7 pg/mL vs 202.0 pg/mL; *P* ≤ .0001; iron-replete, 1.0 pg/mL vs 114.0 pg/mL; *P* ≤ .001; *n* = 8-13). TXA had no effect on IL-6 in either group (Supplementary Figure S10).

## 4 Discussion

Using an established mouse model employing liver laceration and shock [31], we demonstrated that IDA substantially increased traumatic bleeding and mortality. Both bleeding and mortality could be reduced significantly by either correcting the anemia with parenteral iron 2 weeks prior to the traumatic injury or by injecting TXA in the anemic state just before injury. The time point for TXA injection was chosen to mimic peritraumatic administration for the prevention, rather than the treatment, of bleeding complications. Interestingly, elevated platelet counts and FVIII levels in anemic mice did not provide enhanced bleed protection. Reasons may be inherent to platelet dysfunction in the setting of trauma [[43], [44], [45], [46], [47]] and FVIII depletion during TIC.

Following trauma, TIC (evidenced by aPTT prolongation with selective depletion of FV; FVIII; and, partially, fibrinogen) [31,39,40,[48], [49], [50]], pronounced thrombin generation (TAT formation), and pronounced fibrinolysis (PAP and D-dimer formation) developed in anemic and nonanemic mice. However, the survival rate for nonanemic mice was high (∼80%) despite the coagulopathy and marked traumatic blood loss of ∼300 to 500 μL (in relation to total blood volume of ∼1000-1500 μL). Therefore, survival in nonanemic mice in this model could not be improved further with TXA, although TXA prevented excessive bleeding as well as the development of TIC and fibrinolysis. Murine dosing was performed to simulate human dosing in trauma (1 g intravenously [∼10-20 mg/kg]). This dose (10 mg/kg) was used safely previously in a mouse model for traumatic brain injury showing survival benefit [51,52]. While there are no pharmacokinetic data published for mice, we assumed that elimination would be similar to human trauma patients, where most had TXA serum levels of 20 μg/mL at 1 to 2 hours after administration, considered sufficient to inhibit fibrinolysis [53]

In contrast, the survival in anemic mice was much lower (∼50%) and was accompanied by significantly more bleeding and more fibrinolysis than in nonanemic mice. Interestingly although TXA abolished excess bleeding, corrected TIC and thrombin generation, and improved survival to ∼80% (comparable with nonanemic mice), TXA had no effect on fibrinolysis inhibition in the presence of anemia. These observations suggest that pretraumatic anemia predisposes to and/or aggravates systemic excessive fibrinolysis following trauma and that the fibrinolysis developed independently and not as a consequence of TIC and/or bleeding. Excessive fibrinolysis in anemic mice was characterized by pronounced PAP and D-dimer formation. aPTT prolongation despite TXA-mediated TIC correction was due to excessive fibrin split products interfering with aPTT correction as previously described [54]. The susceptibility to excessive fibrinolysis in the anemic state aligns with other studies demonstrating the physiological significance of erythrocytes for clot formation and stabilization. For instance, it has been demonstrated that erythrocytes are needed to stabilize blood clots by providing a packed array of polyhedral structures compressed by a meshwork of fibrin and platelet aggregates [55,56] and by forming projections that strengthen the clot structure [57]. It has also been shown that the fibrin network structure is influenced by erythrocyte concentrations [58,59] and that integration of erythrocytes in clots decreases susceptibility to tissue plasminogen activator (tPA)–induced plasminogen activation, fibrinolysis, and permeability [57]. Moreover, the lack of erythrocytes in fibrin clots reduces the inhibitory potency of TXA significantly [60]. Therefore, it is conceivable that compromised clot stability in the presence of anemia, particularly IDA, with a relative lack of normally shaped erythrocytes (deformed, microcytic, and hypochromic) may enhance tPA access to a loose fibrin mesh causing excessive fibrinolysis unresponsive to TXA. Also, urokinase plasminogen activator rather than tPA-mediated fibrinolysis may be considered where TXA would enhance rather than reduce fibrinolysis [61]. The transition from tPA-mediated to urokinase plasminogen activator–mediated fibrinolysis usually occurs during later stages of trauma with the depletion of plasmin inhibitors and hyperfibrinolysis [62], which has been postulated as a reason for detrimental outcomes and increased bleeding when TXA administration is delayed [63,64]. To that end, it is possible that anemia may impact the timing of this transition given the observed early hyperfibrinolysis with anemia. Another possibility may be inherent to insufficient TXA plasma levels relative to fibrinolysis excess with anemia [65].

However, excessive fibrinolysis did not appear to contribute to mortality in the anemic mice, which was unexpected since hyperfibrinolysis following trauma has been associated with increased mortality in humans [66]. Other than in a controlled mouse model, methods of fibrinolysis determination, type of trauma, resuscitation measures, time of onset, and many other factors may play a role. Notwithstanding these apparent differences, our observations show clearly that reduction of traumatic bleeding through early prevention of TIC (particularly through restoration of FV activity for the prothrombinase complex [31]) by TXA was critical for survival and very efficacious in anemic mice despite the development of excessive systemic fibrinolysis.

We also sought to investigate if TXA exerted anti-inflammatory effects to further explain survival benefits beyond bleed reduction and correction of TIC. There is precedence to support that suppression of inflammation improves survival after trauma, shown in rat models of trauma and hemorrhagic shock [67,68]. Lately, there has been an increasing interest to study potential anti-inflammatory properties of TXA, based on TXA interference with plasminogen binding to inflammatory cells and inhibition of proinflammatory effects exerted by plasmin [69]. In the surgical setting, presurgical IL-6 elevation or the postoperative rise of various inflammatory markers has been associated with adverse outcomes and/or survival [70]. TXA appears to reduce postsurgical levels of IL-6, C-reactive protein, and other selected anti-inflammatory markers with associated improved outcome measures [68,[71], [72], [73]]. Therefore, we measured an array of inflammatory cytokines at baseline, 60 minutes, and 6 hours after trauma. The 6-hour time point was presumed as ideal for cytokine changes in relation to mortality onset (between 8 and 10 hours after trauma). Among the 14 cytokines tested, only IL-6 was increased significantly over baseline in anemic and nonanemic mice at 6 hours (unaffected at 60 minutes). TXA reduced IL-6 levels in both groups of mice, with significance reached in anemic mice. We therefore speculate that the increased bleeding in anemic mice was associated with more pronounced sequelae of hemorrhagic shock associated with evolution of higher IL-6 levels and susceptibility to unabated inflammation, contributing to death [67,74]. In fact, IL-6 concentrations during the first 24 hours following trauma predict organ failure and mortality in trauma patients [75]. Since TXA was able to decrease blood loss and IL-6 levels in anemic and nonanemic mice to similar thresholds (median blood loss, ∼15 μL/g; median IL-6, ∼95 pg/mL), there seems to be a reason to believe that TXA may have influenced survival in anemic mice favorably by curbing inflammatory sequelae of pronounced hemorrhagic shock.

Although this study relates to trauma, the demonstration of increased bleeding in the setting of anemia, which was preventable by TXA administration or correction of anemia with iron supplementation, could also be relevant to similar situations such as surgical interventions and PPH.

One potentially important but unexplained finding was the discovery of elevated FVIII and VWF levels in mice with IDA. A predisposition to VTE in patients with hereditary hemorrhagic telangiectasias (who often suffer from severe iron deficiency due to constant bleeding) has been reported and has been associated with elevated FVIII levels together with low serum iron [76]. There are several other studies suggesting a connection between IDA and VTE [[77], [78], [79], [80]]. One study demonstrated that intravenous iron treatment reduces coagulability [81]. To the best of our knowledge, direct evidence of elevated FVIII and VWF in conjunction with iron deficiency has not been reported before, and neither has potential beneficial effects on bleeding propensity in anemic states. While the etiology of this finding is unexplained and requires further study, this finding may explain the heightened VTE risk observed in IDA [82].

## 5 Conclusion

In summary, our findings provide proof-of-principle that IDA increases acute bleeding and mortality after trauma, both of which could be rescued by prophylactic TXA. These findings support immediate peritraumatic TXA administration, which may be particularly lifesaving in low- and middle-income countries where IDA is highly prevalent and traumatic deaths are disproportionally high than in high-income countries. In addition, these findings should provide impetus for studies where anemia can be recognized and corrected in timely relation to expected bleeding, such as PPH or surgical interventions, or where TXA can be administered prophylactically when anemia is present.
