# Supplementary material for: Traumatic bleeding and mortality in mice are intensified by iron deficiency anemia and can be rescued with tranexamic acid
Source: Res Pract Thromb Haemost. 2024 Aug 8;8(6):102543. doi: 10.1016/j.rpth.2024.102543 (PMC11403369; doi:10.1016/j.rpth.2024.102543)
Supplement: Supplementary material [file mmc1.docx]

**Supplementary Information**

**Traumatic bleeding and mortality in mice are intensified by iron deficiency anemia and can be rescued with tranexamic acid prophylaxis**

Bilgimol Chumappumkal Joseph, Tro Sekayan, Nicca Falah, Richard F W Barnes, Veronica Flood, Juan A. De Pablo-Moreno, Annette von Drygalski

T = -5 minutes

**INTERVENTION**

TXA bolus

(Prophylaxis)

T = 0 minutes

**TRAUMA**

Liver laceration

T = 60 minutes

**Severe Bleeding**

**Surgical Closure**

**Observation Mortality Start**

T = 60 minutes and 6 hours

**ANALYSIS**

Cytokine panel

T = 15 minutes

**ANALYSIS**

Blood loss, APTT,

coagulation factors,

TAT, and PAP-complexes, D-dimer

**Supplementary Figure S1. Experimental set-up and timeline of the liver laceration model.** Mice were subjected to severe bleeding by midline laparotomy followed by liver laceration involving the removal of ~75% of the left liver lobe. Mice received vehicle control (saline 100 µL) or TXA (10 mg/kg) as a bolus intravenous administration 5 minutes before liver laceration. Pre-weighed sponges were inserted into the abdominal cavity prior to liver laceration. Wound clips were used to close the abdomen and blood-soaked sponges were collected and weighed 60 minutes after liver laceration to determine blood loss. Blood was collected by cardiac puncture or by retroorbital 60 minutes and 6 hours after liver laceration. Plasma samples were tested for aPTT, coagulation factors, TAT, PAP complexes, and D-Dimer at 60 minutes, and at 60 minutes and 6 hours for cytokines, respectively. aPTT, Activated partial thromboplastin time; D-Dimer, D fragment of fibrin; PAP, plasmin-alpha-2-antiplasmin; TAT, Thrombin anti-thrombin; TXA, Tranexamic Acid; T, Time.


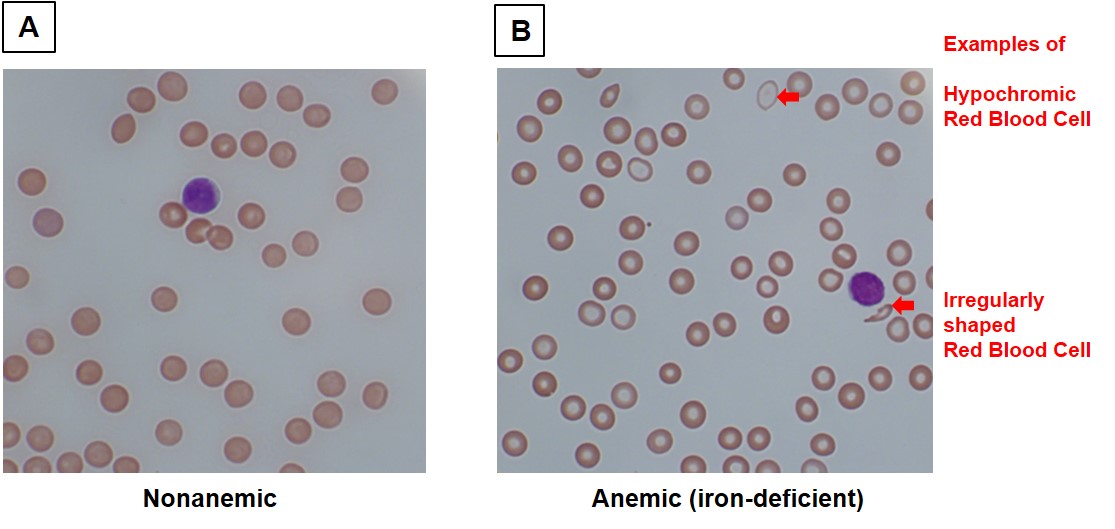


**Supplementary Figure S2. Peripheral blood smears.** Peripheral blood smears from nonanemic and anemic mice (C57BL/6J) were prepared at 6 weeks of age (Wright-Giemsa staining). The digital photomicrographs were taken using an Olympus BH2 microscope and an oil immersion lens at 100x equipped with an Olympus MicroFire digital camera. Shown are representative examples of red cells of (A) nonanemic and (B) anemic mice. Microcytic, hypochromic, and irregularly shaped red blood cells are characteristic of iron deficiency anemia and examples are marked by red arrows.


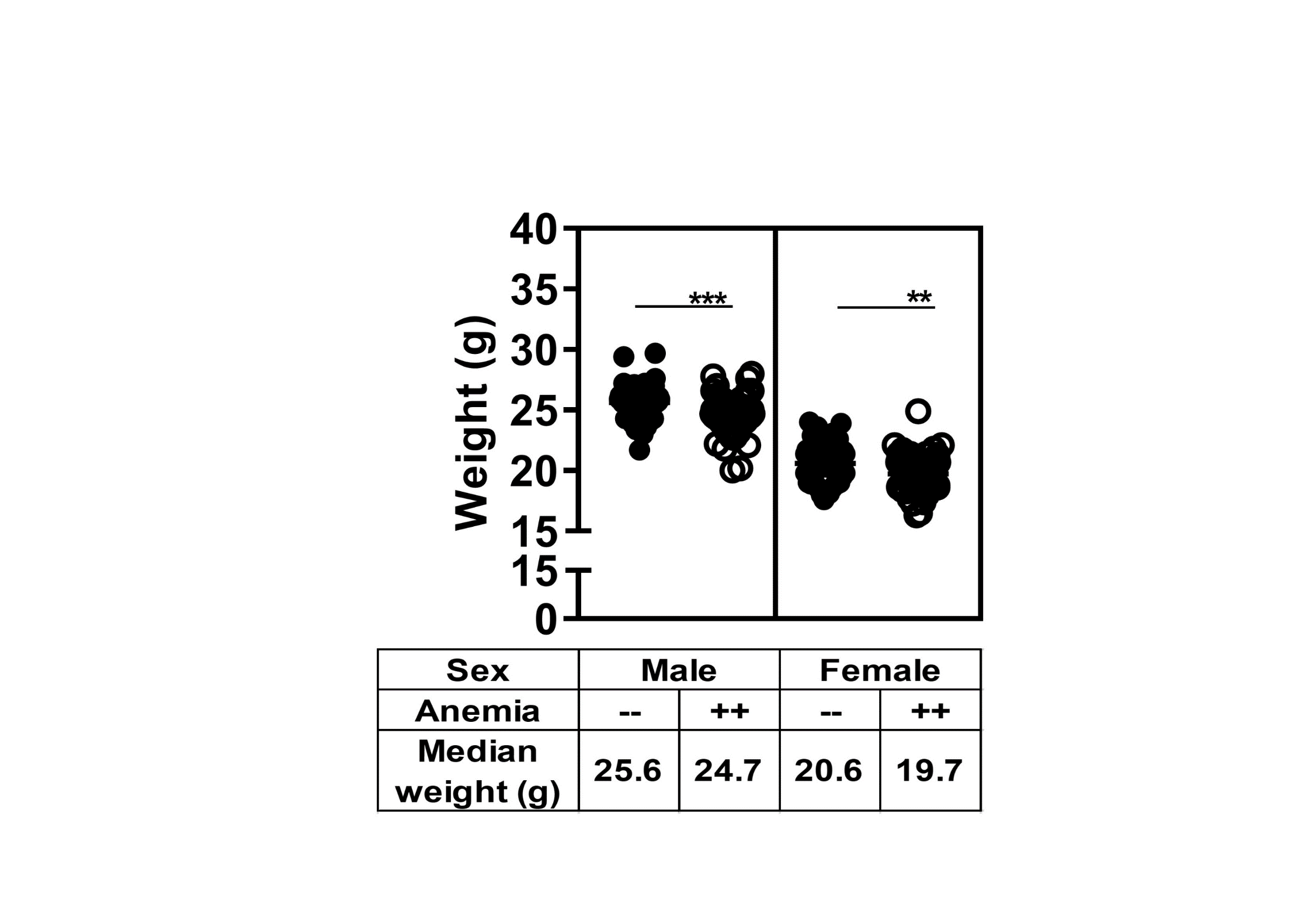


**Supplementary Figure S3. Mouse body weight.** Mice were weighed prior to liver laceration (n=55 per group). Nonanemic mice were compared to anemic mice of the same sex with horizontal bars showing the median. Samples were compared with non-parametric Mann-Whitey test comparing non-anemic to anemic mice in each sex group (**p≤0.01, ***p ≤0.001).


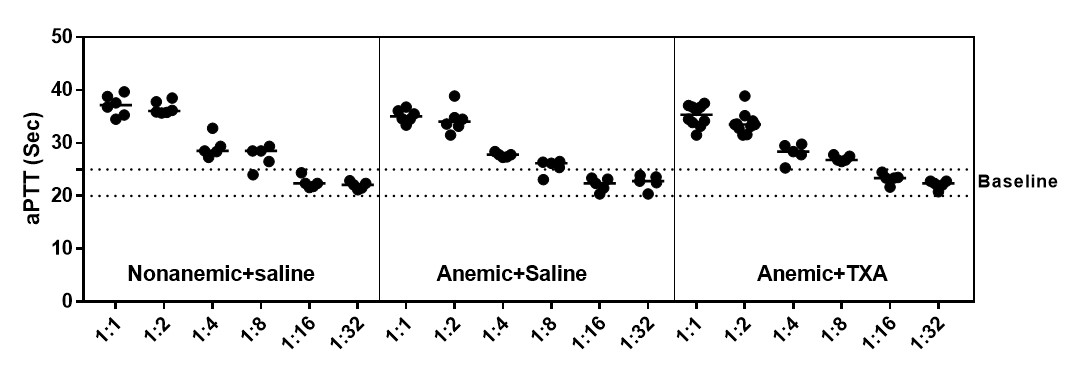


**Supplementary Figure S4. aPTT correction with normal plasma after liver laceration**. Plasma aPTT level was determined at 1 hour after induction of severe bleeding by a midline laparotomy followed by LL in mice (n=5-10). Mice were pre-treated either with saline (nonanemic and anemic) or with TXA (anemic) (10mg/kg). Serial 1:1 dilution (1:1, 1:2, 1:4, 1:8, 1:16, and 1:32) of test plasma with normal mouse plasma was performed followed by immediate aPTT assessment. The horizontal bars show the median. Bl, baseline; LL, Liver laceration; TXA, Tranexamic Acid


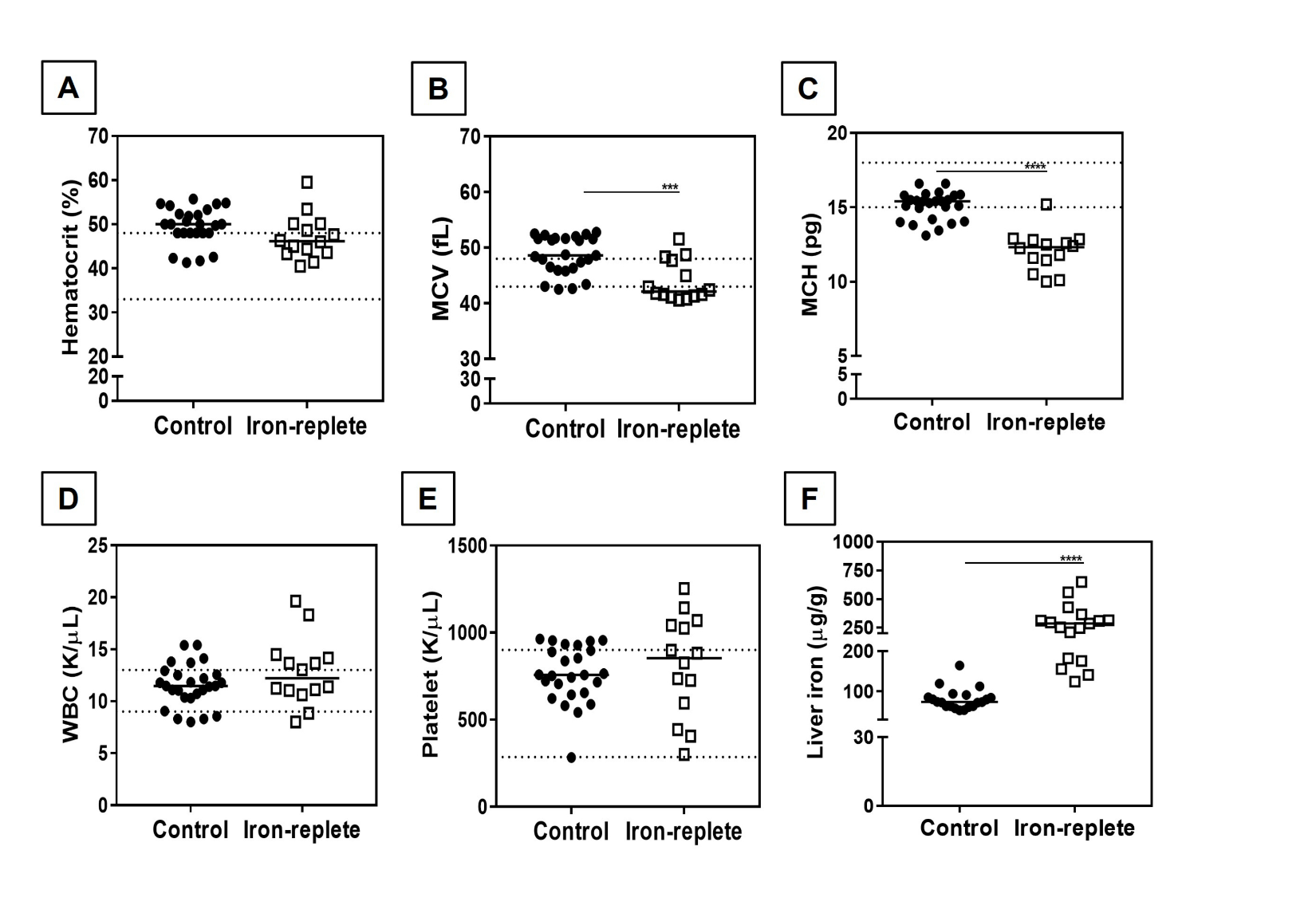


**Supplementary Figure S5. CBC and liver iron content in iron-replete mice.** Prior to LL, CBC and liver iron content were analyzed in iron-replete mice (iron-deficient diet with parenteral iron treatment 2 weeks prior to LL). Results were compared to control mice fed with a normal diet. (A) Hematocrit, (B) MCV, (C) MCH, (D) WBC, (E) platelets, and (F) liver iron. Samples were compared with the non-parametric Mann-Whitney test (n=10-15). The horizontal bars represent the median (***p≤0.001, ****p≤0.0001). The area between the dotted lines marks the normal range for C57Bl/6 mice. CBC, Complete blood count; LL, Liver Laceration; MCH, mean corpuscular hemoglobin; MCV, mean corpuscular volume; WBC, white blood cells.


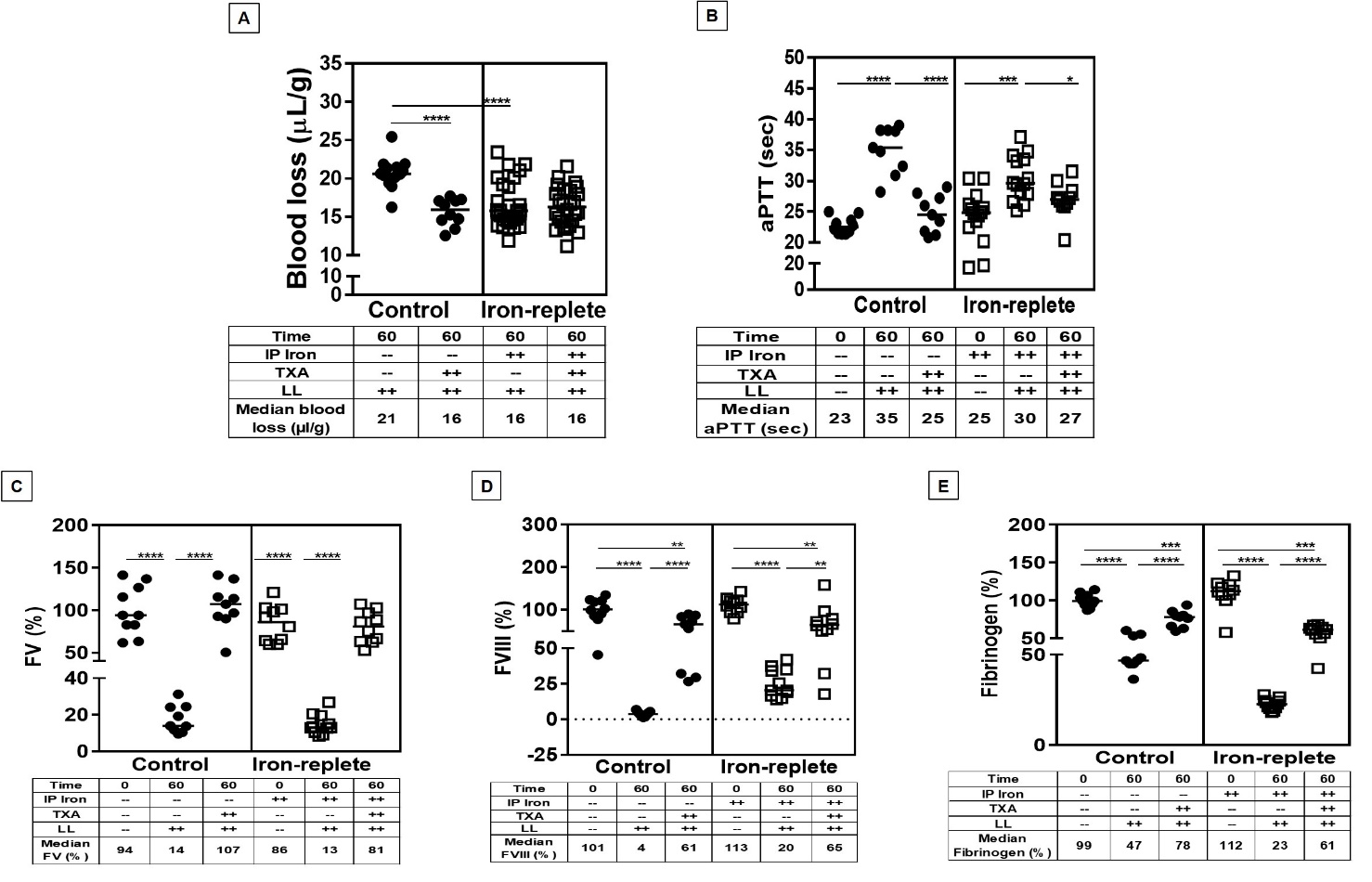
**Supplementary Figure S6. Blood loss and coagulopathy after liver laceration in iron-replete mice.** Severe bleeding was induced by a midline laparotomy followed by LL. Control and iron-replete mice (iron-deficient diet with parenteral iron treatment 2 weeks prior to LL) were treated prophylactically with 100 µL of saline or TXA (10 mg/kg). Blood loss and coagulation parameters were determined in each group at 60-minute time points. (A) Blood loss, (B) aPTT, (C) FV, (D) FVIII, and (E) Fibrinogen (n=7-21). Samples were compared with the non-parametric Mann-Whitney test. Results were compared to the baseline with horizontal bars showing the median (*p≤0.05, **p≤0.01, ***p≤0.001, ****p≤0.0001). aPTT, Activated partial thromboplastin time; FV, Factor V; FVIII, Factor VIII; IP, intraperitoneal; LL, Liver Laceration; TXA, Tranexamic Acid.


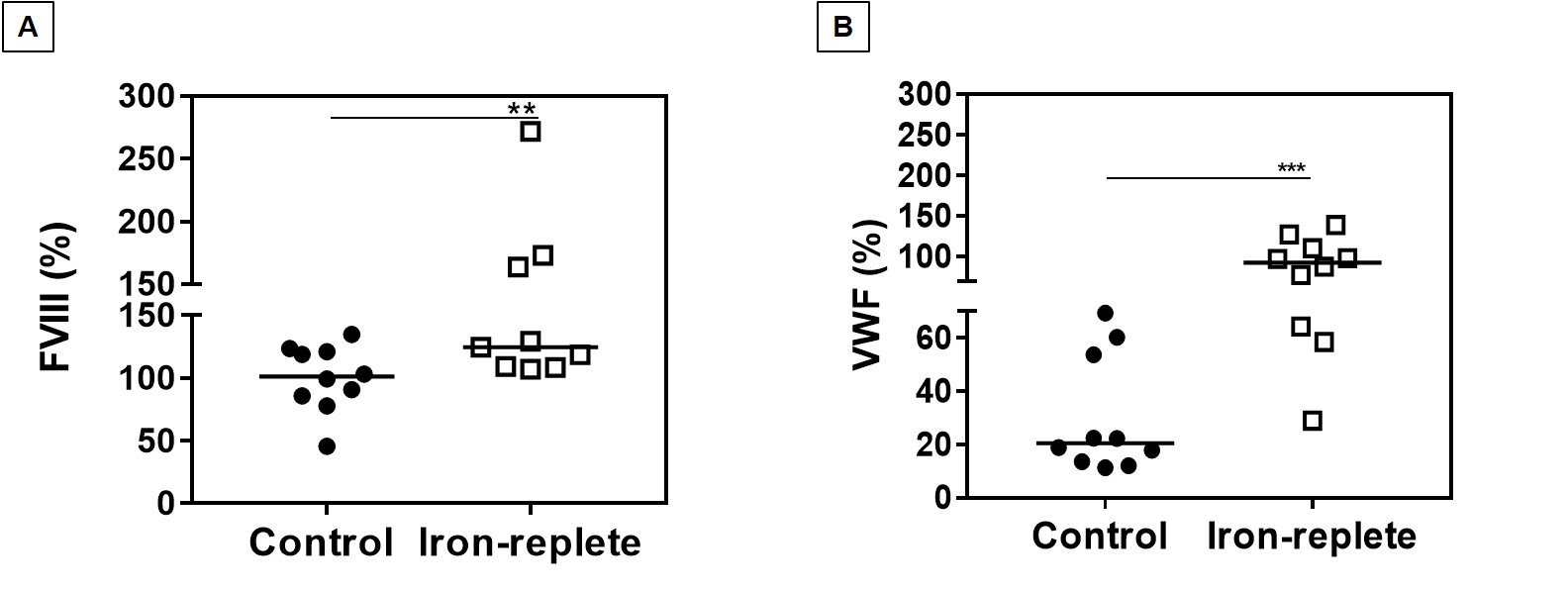
**Supplementary Figure S7. FVIII and VWF levels in iron-replete mice.** Baseline plasma samples of control and iron-replete mice (iron-deficient diet with parenteral iron treatment 2 weeks prior to LL) were analyzed for (A) FVIII levels by chromogenic assay and (B) VWF antigen levels by ELISA (n=9-10 per group). (**p≤0.01, ***p≤0.001). ELISA, Enzyme-linked immunoassay; FVIII, Factor VIII; LL, liver laceration; VWF, Von Willebrand Factor.


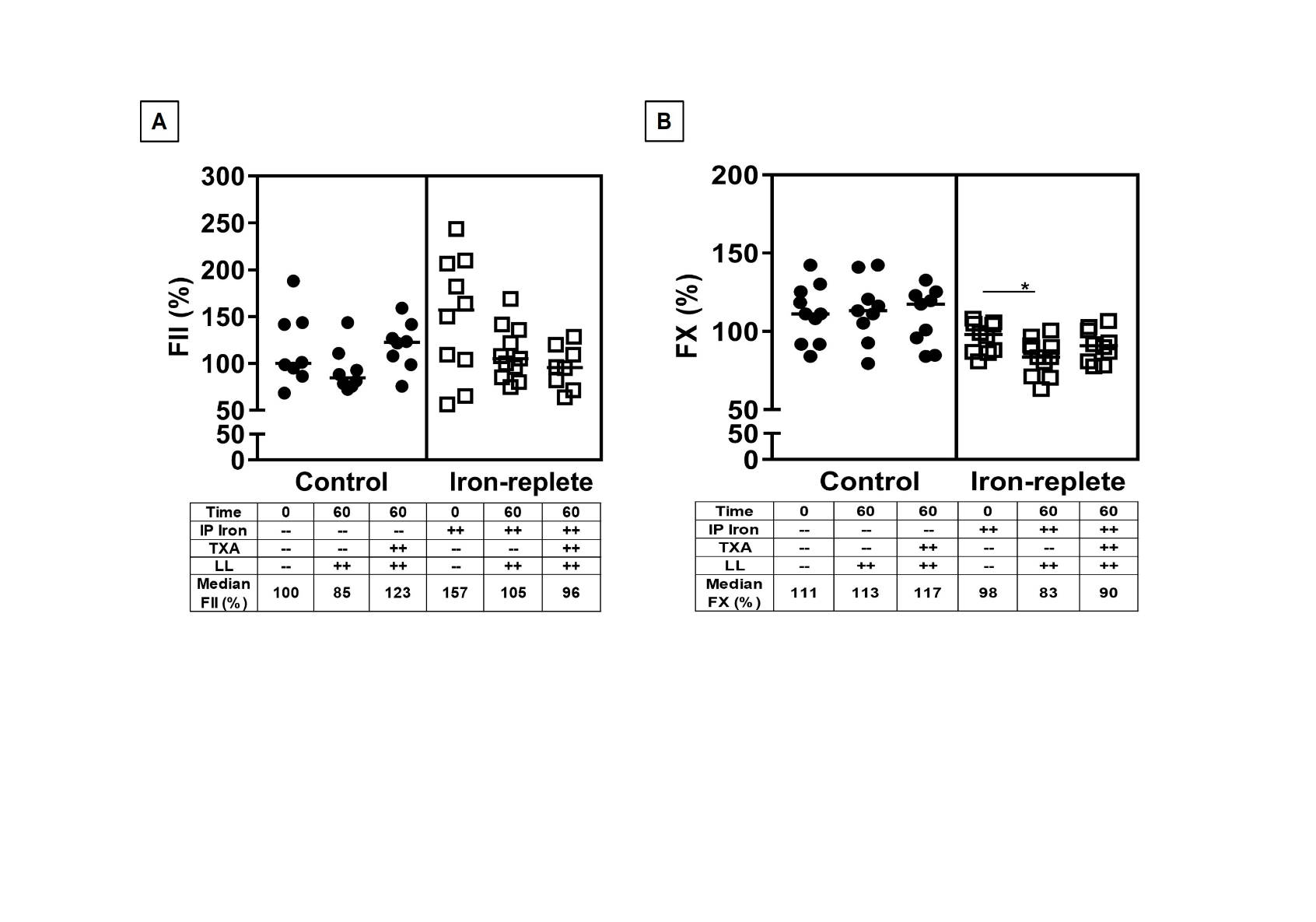


**Supplementary Figure S8. FII and FX after liver laceration in iron-replete mice.** Severe bleeding was induced by a midline laparotomy followed by LL. Control and iron-replete mice (iron-deficient diet with parenteral iron treatment 2 weeks prior to LL) were treated prophylactically with 100 µL of saline or TXA (10 mg/kg). FII and FX were determined in each group at 60-minute time points. (A) FII, (B) FX, (n=7-21). Samples were compared with the non-parametric Mann-Whitney test. Results were compared to the baseline with horizontal bars showing the median (*p≤0.05). FII, Factor II; FX, Factor X; IP, intraperitoneal; LL, Liver Laceration; TXA, Tranexamic Acid.


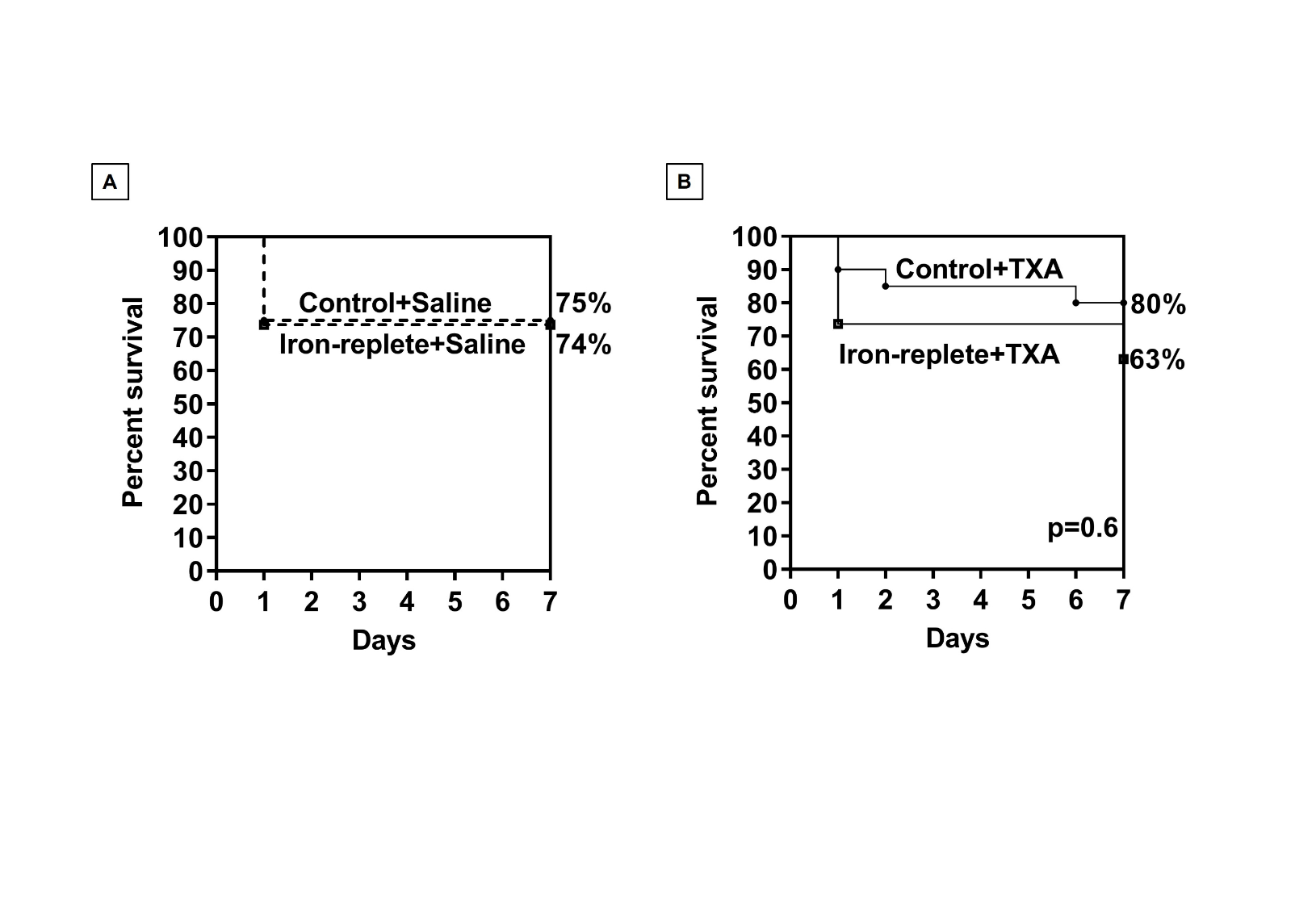


**Supplementary Figure S9. Iron repletion and survival after liver laceration**. 7-day survival of control and iron-replete mice after trauma was determined by Kaplan-Meyer analysis. Mice underwent midline laparotomy with subsequent liver laceration and were treated prophylactically with 100 µL of saline or TXA (10 mg/kg) five minutes prior. After blood loss determination was completed (60 minutes after trauma), wounds were closed and the mice were returned to the cages, given 400 µL of saline for the first 3 days, and monitored 7 days for survival (n=20 mice per group). (A) control mice and iron-replete with no treatment and (B) control and iron-replete mice treated with TXA. TXA, Tranexamic Acid.


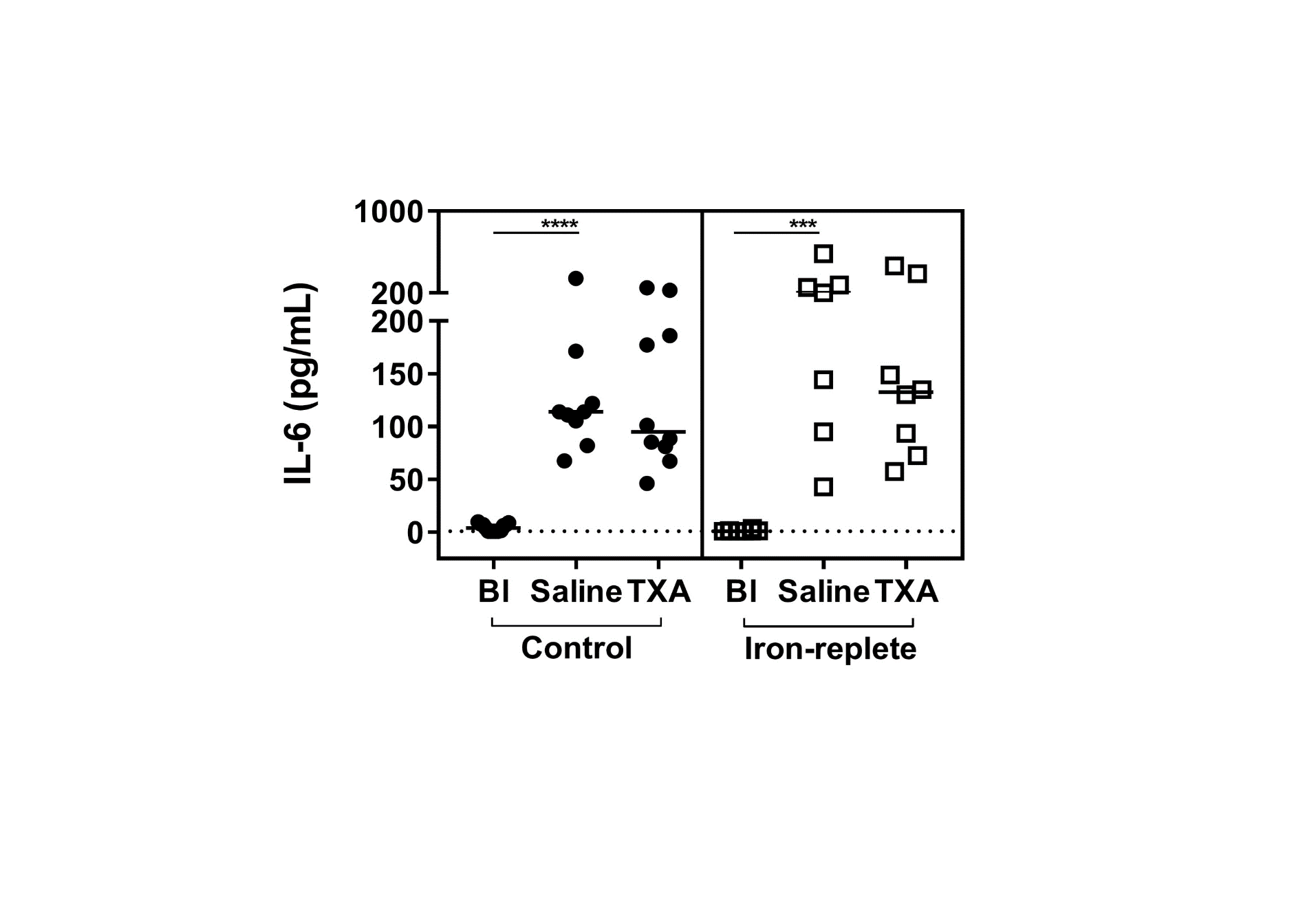


**Supplementary Figure S10. Inflammatory effects after liver laceration in iron-replete mice**. Plasma IL-6 level was determined at baseline and 6 hours after induction of severe bleeding by a midline laparotomy followed by LL in control and iron-replete mice (iron deficient diet with parenteral iron treatment 2 weeks prior to LL) (n=8-12). Samples were compared with non-parametric Mann-Whitney test. Results were compared to baseline with horizontal bars showing the median (***p≤0.001, ****p≤0.0001). Bl, baseline; IL-6, interleukin-6; LL, Liver laceration; TXA, Tranexamic Acid.
